# Radicalization Leading to Violence: A Test of the 3N Model

**DOI:** 10.3389/fpsyt.2019.00042

**Published:** 2019-02-11

**Authors:** Jocelyn J. Bélanger, Manuel Moyano, Hayat Muhammad, Lindsy Richardson, Marc-André K. Lafrenière, Patrick McCaffery, Karyne Framand, Noëmie Nociti

**Affiliations:** ^1^Department of Psychology, New York University Abu Dhabi, Abu Dhabi, United Arab Emirates; ^2^Department of Psychology, University of Cordoba, Cordoba, Spain; ^3^Department of Psychology, University of Peshawar, Peshawar, Pakistan; ^4^College of Social and Behavioral Sciences, Walden University, Minneapolis, MN, United States; ^5^Department of Psychology, McGill University, Montreal, QC, Canada; ^6^Department of Psychology, Carleton University, Ottawa, ON, Canada; ^7^Department of Psychology, University of South Australia, Adelaide, SA, Australia; ^8^Department of Psychology, Université du Québec à Montréal, Montreal, QC, Canada

**Keywords:** social alienation, support for political violence, radicalization, violent extremism, 3N model of radicalization

## Abstract

The present research examines the social cognitive processes underlying ideologically-based violence through the lens of the 3N model of radicalization. To test this theory, we introduce two new psychometric instruments—a social alienation and a support for political violence scale—developed in collaboration with 13 subject matter experts on terrorism. Using these instruments, we test the theory's hypotheses in four different cultural settings. In Study 1, Canadians reporting high levels of social alienation (Need) expressed greater support for political violence (Narrative), which in turn positively predicted wanting to join a radical group (Network), controlling for other measures related to political violence. Study 2a and 2b replicated these findings in Pakistan and in Spain, respectively. Using an experimental manipulation of social alienation, Study 3 extended these findings with an American sample and demonstrated that moral justification is one of the psychological mechanisms linking social alienation to supporting political violence. Implications and future directions for the psychology of terrorism are discussed.

## Introduction

The threat of terrorism is a global concern. Despite declining numbers of terrorist attacks worldwide in the last 3 years, violent extremism “remains extraordinarily high compared to historical trends” [(1), p. 1]) with more than 10,000 terrorist attacks claiming the lives of over 25,000 people annually (2). What has also been noticeable is the sheer brutally of these incidents such as the 2014 Gamboru Ngala massacre in Nigeria (more than 300 deaths), the 2015 Bataclan attack in France (killing 137 and injuring more than 400 people), and the 2016 Karrada bombing in Iraq (killing more than 300 people and injuring over 200). The frequency and intensity of violent attacks committed by widely different ideological groups (e.g., far-right and far-left movements, radical Islam) has motivated the creation of several local anti-radicalization programs (e.g., Calgary's Redirect initiative, Montreal's Centre for the Prevention of Radicalization Leading to Violence) and national strategies to tackle the rise of homegrown terrorism [see (3)].

In light of these global trends, one pressing question for psychologists is why are young adults impelled to join violent organizations? One view holds that social alienation—a state of estrangement and detachment from society— is an important vulnerability that prompts individuals to seek solace in radical groups (4–6) that promise camaraderie and purpose to those that follow their ideological imperatives (7). Accordingly, disenfranchised and alienated individuals are assumed to be at risk of becoming terrorism recruits, especially if there are no alternative outlets to their frustration (8); see (9, 10).

In spite of the intuitive appeal of the social alienation hypothesis, an exhaustive literature search (using PsychINFO and EBSCO databases) indicates that evidence linking social alienation, support for violence, and wanting to join a violent group is lacking. To date, the most relevant piece of research has been on marginalization: “a condition wherein individuals do not identify with either the home or the host culture” [(11), p. 3]. In a cross-sectional investigation of first and second-generation Muslim immigrants in the US, the authors found that marginalization was positively related to significance loss (i.e., low self-worth), which, in turn, positively predicted support for (1) radical interpretation of Islam and (2) fundamentalist groups (11). The present research builds on this important piece of research and contributes to this literature by making the following distinctions:

First, extending the work of Lyons-Padilla et al. (11) we posit that minorities within society that struggle to reconcile conflicting cultural identities are not the only members of society at risk of being attracted to violent extremism. As we intend to demonstrate, members of the host culture, too, can feel detached from society and embrace political violence with great resolve and seek the company of those that share a similar predisposition.

Second, we postulate that social alienation, support for political violence, and support for violent groups are interwoven components of a motivational process that characterizes a specific trajectory toward violence. As we lay out in the following sections, the present research examines the social cognitive processes underlying ideologically-based violence through the lens of the 3N model of radicalization (7, 12, 13); a new theoretical perspective which warrants further investigation. To test this theory, we introduce two new instruments developed in collaboration with 13 subject matter experts on terrorism: A scale that measures social alienation, people's detachment from society, and a scale that measures support for political violence, people's proclivity to accept violence to further an ideological cause.

Third, we adduce cross-cultural evidence for the aforementioned motivational process and examine more closely the cognitive mechanism that facilitates supporting political violence when social alienation is experimentally induced by investigating the role of moral disengagement (14). The present research extends the 3N model of radicalization and provides a comprehensive understanding of radicalization to violence by combining insights from multiple theoretical perspectives.

## The 3N Model of Radicalization

Radicalization is commonly defined as “the social and psychological process of incrementally experienced commitment to extremist political or religious ideology” [(15), p. 152]. The 3N model of radicalization (12) identifies *need, narrative*, and *network*, as the three categories of factors involved in producing radicalization toward violence. We cover them in turn and discuss how they are dynamically related.

### Need

One perennial question among terrorism researchers has been what motivates individuals to become involved in violent extremism. According to Kruglanski et al. (7, 13, 16, 17), one motivational impetus underlying radicalization is the “quest for significance,” namely, the universal *need* to be someone and to be respected by others that matter. The significance quest usually becomes an important goal when significance is lost (7) such as when people experience personal failure, rejection, and humiliation (18).

Social alienation—the feeling of detachment from social and cultural participation (19)—is another form of significance loss that has been discussed in many theories of radicalization [e.g., (20–23). The literature on social alienation suggests that it is a “sign of personal dissatisfaction with certain structural elements of society…related particularly to economic and political elements” [(24), p. 90]. It is a psychological state that stems from feeling ostracized [for a review see (25)] and not having genuine bonds with others, but it can also be actively sought by rejecting society's values and excluding oneself from the community (26–28). As a result, socially alienated individuals feel disconnected from the values of society and other citizens (29, 30), are typically less interested in public affairs, and do not identify with political figures (31).

Research by Shmotkin and Litwin (32) suggests that social alienation is a devastating feeling that is strongly associated with loss of personal worth and purpose. The 3N model of radicalization proposes that when significance is lost, individuals become motivated to restore it (18). One way to restoring significance is by retaliating against the source of the threat or seeking out new groups that can provide camaraderie and purpose (5, 33). In line with this proposition, Wiktorowicz (6) has suggested that negative personal events such as social alienation induces a “cognitive opening,” a receptiveness to new, and potentially violent, ideologies [see (10)]. Similarly, it has been proposed that individuals feeling consistently neglected and living on the fringe of society can be potential recruits for terrorism, especially if there is no outlet for their frustration (8–10).

Several historical cases suggest that social alienation may have been a driving force behind radicalization leading to violence. One of the most documented examples is that of the Chechen Black Widows, a female-dominated group that sought to avenge the deaths of their husbands and families at the hands of Russian troops. According to Speckhard and Akhmedova's (34) analysis, soon after this traumatic event, 92% of Chechen female suicide bombers (24/26) experienced social alienation and 73% “sought a connection to Wahabbists groups soon after the trauma and in direct reaction to it” (p. 5).

### Narrative

As exemplified by the Black widows, one common response to experiencing a painful loss of significance (e.g., frustration, humiliation) is wanting to punish those responsible for one's suffering [see (35, 36)]. Although displaying power through forceful and heavy-handed behavior may be instrumental to reassert one's significance, the use of violence is generally prohibited and socially reprimanded. However, violence becomes permissible when it is encapsulated within an ideological framework that provides moral justifications for its use against a specific group of people.

This suggests that ideological narrative play two important roles. On the one hand, ideologies are shared systems of belief (37) that identify the actions required to achieve significance, which typically involves extreme violence against the perceived enemies of one's (ethnic, religious, or social) group. On the other hand, ideological narrative provides the moral justifications rendering violence acceptable and even desirable against outgroup members (7, 14, 38).

This echoes the work of Bandura (14) on moral disengagement that has marshaled evidence for the notion that people engage in many cognitive maneuvers to proceed with unethical behaviors without self-recrimination. For example, unethical behavior appears permissible when victims are *dehumanized* (considered lesser beings) and when violence is *morally justified* by portraying it as a noble and important cause. Supporting that claim, Aquino et al. (39) found that “moral disengagement effectively reduced the extent to which participants experienced negative emotions in reaction to abuses of Iraqi detainees by American soldiers” (p. 385). In the present research, we make the prediction that because socially alienated individuals are not meaningfully connected to their community, they should be more inclined to be morally disengaged and thus, more prone to support political violence. This hypothesis is consonant with research showing that feeling disconnected from others reduces empathy, prosocial behavior, and increases aggressive behavior (40, 41).

### Network

Once people adhere to the ideological narrative that morally justifies the use of violence to restore significance, people are likely to be motivated to seek the presence of others that share similar beliefs. People's beliefs are likely to influence the type of group they join because of the universal motivation to attain mutual understanding and a shared sense of reality (42–44). These epistemic and relational motives are satisfied by obtaining a predictable and controllable environment in a group of like-minded individuals (37). Thus, those supporting violence are likely to seek the company of individuals sharing similar ideological beliefs. Furthermore, by joining a group of like-minded individuals, the use of violence is socially condoned as those that defend the existence of the group are bestowed significance and referred to as heroes and martyrs (7, 13, 16).

## The Present Research

As the present literature review attests, there is evidence suggesting that taken separately, Need, Narrative, and Network can fuel support for political violence. However, what is unique about the 3N model of radicalization is that it postulates that these three ingredients are dynamically interconnected. Specifically, one radicalization trajectory postulated by the theory is the transition from Need, Narrative, to Network—i.e., from individuals experiencing a loss of significance (e.g., social alienation), to believing that violence is an acceptable way to restore that loss, to wanting to join a like-minded group. The purpose of this research was to test these postulates.

To that end, in Study 1 we created two new psychometric instruments: one to measure social alienation and one to measure support for political violence. In a Canadian sample, we predicted that social alienation (Need) would be positively associated with support for political violence (Narrative) as a means to address their grievance, which in turn would be associated with wanting to join like-minded others (Network) that support violent methods of achieving their political goals. Building on previous work, we predicted this radicalization trajectory would hold above and beyond other known predictors of violent extremism. Specifically, we controlled for people's (1) willingness to self-sacrifice for a cause (45); (2) collective narcissism [convictions about the superiority of one's in-group; (46)], and (3) need for cognitive closure (47, 48).

In Study 2a and 2b, we aimed to replicate this model in two different cultural contexts, namely, Pakistan and Spain. In Study 3, we aimed to experimentally replicate Study 1, 2a, and 2b by manipulating social alienation and examining its influence on support for political violence. The purpose of Study 3 was also to test the mediating role of moral disengagement. Distinguishing between moral justification and dehumanization, two strategies typically related to supporting violence (14), we predicted that people that feel socially alienated would be more prone to morally justify violence and dehumanize others, which in turn would make them more likely to support political violence.

## Study 1

The purpose of Study 1 was to adduce empirical evidence for the psychological trajectory postulated by the three Ns model of radicalization. In line with this theoretical framework, we expected that social alienation would be positively associated with support for violence, which in turn would be associated with wanting to join a radical group. We aim to provide support for this model by controlling for variables that been previously associated with political violence, namely, collective narcissism, need for closure, and self-sacrifice.

### Methods

#### Participants and Procedure

Assuming medium effect sizes, six latent variables, 42 observed variables, and power set at 0.80, a sample size of 400 was suggested (49). Four hundred and seventy Canadians (205 women; *M*_*age*_ = 32.67, *SD*_*age*_ = 13.82) were recruited. Written consent was obtained from participants. Participants were recruited via advertisements posted on classified advertisements, social media, and Amazon's Mechanical Turk.

### Material

#### Item Development

A total of 56 items were developed for the Social Alienation scale and 54 for the Support for Political Violence scale. Participants indicated the extent to which they agreed with these statements using a 6-point Likert scale ranging from 1 (*strongly disagree*) to 6 (*strongly agree*).

After the items were developed, 13 independent subject matter experts^1^ (SMEs) were consulted to verify the content validity of the potential scale items. Content validity is the degree that a subject's response on a test represents their responses to a real situation, constituting the area of concern for the interpreters of the test (50). This form of external validation is consistent with the goal of producing reliable findings (51, 52). The SMEs possessed a breadth and depth of knowledge, spanning frontline, operational, scholarly, and theoretical perspectives on terrorism. SMEs completed standardized content validity forms developed by DeMaio and Landreth (53) to identify problems with questions that may impact data quality.

#### Collective Narcissism

To measure collective narcissism, three items (Study 1a: *a* = 0.84; *M* = 2.79, *SD* = 1.31, Study 1b: α = 0.79; *M* = 2.75, *SD* = 1.17) were adapted from the Collectivism Narcissism scale proposed by de Zavala et al. (46). A sample item reads, “My group deserves special treatment” and was completed on a 6-point Likert scale ranging from 1 (*strongly disagree*) to 6 (*strongly agree*)—the notion of group was not specifically defined so that participants could define it according to what felt personally relevant.

#### Self-Sacrifice

Readiness to self-sacrifice was measured with the 10-item Self-Sacrifice Scale proposed by Bélanger et al. (45); Study1a: *a* = 0.86; *M* = 3.50, *SD* = 1.28, Study 1b: α = 0.82; *M* = 2.63, *SD* = 1.06). A sample item reads, “I would be ready to give my life for a cause that is extremely dear to me” and was completed on a 7-point Likert scale ranging from 1 *(not agree at all*) to 7 (*very strongly agree*).

#### Need for Closure

Need for closure was measured with Roets and Van Hiel's (54) 15-item Need for Closure Scale (Study 1a: *a* = 0.88; *M* = 3.60, *SD* = 0.56, Study 1b: α = 0.89; *M* = 4.11, *SD* = 0.78). A sample item reads, “I dislike unpredictable situations” and was completed on a 6-point Likert scale ranging from 1 (*strongly disagree*) to 6 (*strongly agree*).

#### Wanting to Join a Radical Group

Participants' proclivity toward joining a radical group was measured with two items on a Likert scale (Study1a: *r*_s_ = 0.79; *M* = 2.89, *SD* = 1.47, Study 1b: *r*_s_ = 0.82; *M* = 1.70, *SD* = 1.05). The items were “I would support a group that is not afraid of defying the law to fight for its principles” and “I would join a group that is willing to use all means possible to defend its ideology” and was completed on a 6-point Likert scale ranging from 1 (*strongly disagree*) to 6 (*strongly agree*).

### Results and Discussion

#### Factorial Validity and Reliability of the Social Alienation Scale

The factorial validity of the social alienation scale was tested by randomly dividing the sample in two groups. The first group was used to conduct an exploratory factor analysis (EFA) and the second group was used to conduct confirmatory factor analysis (CFA). Expectation maximization (55) was used to replace isolated missing values (0.002% of all data) so that a covariance matrix based on the entire sample could be generated for the SEM analysis. The EFA was performed on 235 participants using maximum likelihood and oblimin solution. Items that loaded on multiple factors or with weak factor loading were eliminated (56). We then selected the 6 items with the strongest factors loadings (3 were reverse-coded).

A second EFA (with maximum likelihood and oblimin rotation) was performed with those 6 items. Results indicated a two-factor solution with eigenvalues of 3.87 and 1.12 explaining 64 and 18%, respectively. Items that loaded on the first factor included statements such as “I refuse to be part of Canadian society” and “I identify strongly with Canadian culture and values” (reverse-scored). The oblimin rotation revealed that all the positive items loaded on one factor whereas all the reverse-scored items loaded strongly on the second factor (without cross-loadings). Previous research has shown that loading patterns like these are an artifact of item wording [(57); for a discussion, see (58)]. Thus, we predicted that a unique factor structure would fit the data better than a two-factor solution.

To test these predictions, a CFA was conducted with AMOS (59) using the second random sample of 235 participants. The single factor model was tested with unstandardized coefficients obtained from the maximum-likelihood method of estimation. A model with acceptable fit should have a comparative fit index (CFI) and Tucker-Lewis index (TLI) superior to 0.90, and models with excellent fit should have fit statistics superior to 0.95 (60, 61). Additionally, the root-mean-square error of approximation (RMSEA) and standardized root-mean-square residuals (SRMR) should be 0.08 for acceptable and 0.05 for excellent model fit. Results from the CFA yielded a good fit to the data, χ2_(df = 4)_ = 3.88, *p* = 0.42, CFI = 1.00, TLI = 1.00, RMSEA = 0.00, SRMR = 0.01. Results revealed high levels of reliability for the Social Alienation scale (*a* = 0.87). To ensure that the proposed single factor solution was the best fitting model, it was compared to an alternative two-factor solution whereby the reversed items loaded on one factor and the non-reversed items loaded on another factor. Results indicated that the one-factor solution had a best fit to the data compared to the two-factor solution, $\Delta \chi_{\left(4\right)}^{2}$ = 30.72, *p* < 0.001. See Table 1 for items and factor loadings.

**Table 1:** Final item selection for the Social Alienation Scale (Study 1).

**Items**	**Factor loadings**
I avoid social gatherings and activities associated with Canadian society.	0.81^***^
I refuse to be part of Canadian society.	0.86^***^
I strive to be distant from the average Canadian.	0.82^***^
I fit in well with Canadian values and beliefs (R).	0.49^***^
I have stable and positive interactions with others from Canadian society (R).	0.64^***^
I identify strongly with Canadian culture and values (R).	0.47^***^

*** *p < 0.001, Reverse-scored items = (R)*.

### Factorial Validity and Reliability of the Political Violence Scale

The same procedure was used to test the factorial validity of the Political Violence scale. An EFA was performed with 235 participants using maximum likelihood and oblimin solution. Items that loaded on multiple factors or with weak factor loading were eliminated (56). We then selected the 6 items with the strongest factors loadings (3 were reverse-coded).

A second EFA (with maximum likelihood and oblimin rotation) was performed with those 6 items. Results indicated a two-factor solution with eigenvalues of 3.30 and 1.44 explaining 55 and 24% of the variance, respectively. Items that loaded on the first factor included statements such as “violence is necessary for social change” and “there are effective ways of changing society in Canada other than resorting to violence” (reversed-scored). The oblimin rotation also revealed that all the positive items loaded on one factor whereas all the reverse-scored items loaded strongly on the second factor (without cross-loadings) and we thus predicted that a unique factor structure would fit the data better than a two-factor solution.

To test these predictions, a CFA was conducted using the second random sample of 235 participants. The single factor model was tested with unstandardized coefficients obtained from the maximum-likelihood method of estimation. Results from the CFA yielded a good fit to the data, χ2_(df = 1)_ = 0.09, *p* = 0.76, CFI = 1.00, TLI = 1.00, RMSEA = 0.00, SRMR = 0.002. Results revealed high levels of reliability for the Political Violence scale (*a* = 0.83). To ensure that the proposed single factor solution was the best fitting model, it was compared to an alternative two-factor solution whereby the reversed items loaded on one factor and the non-reversed items loaded on another factor. Results indicated that the one-factor solution had a best fit to the data compared to the two-factor solution, $\Delta \chi_{\left(7\right)}^{2}$ = 19.06, *p* < 0.01. See Table 2 for items and factor loadings.

**Table 2:** Final item selection for the Political Violence Scale (Study 1).

**Items**	**Factor loadings**
When using violence to further a just cause, everybody is fair game.	0.81^***^
Violence is necessary for social change.	0.86^***^
It is acceptable to retaliate against someone who insults my values and beliefs.	0.73^***^
I would never consider physical violence to further a just cause (R).	0.46^***^
We should never use violence as a way to try to change society (R)	0.50^***^
There are effective ways of changing society in Canada other than resorting to violence (R)	0.49^***^

*** *p < 0.001, Reverse-scored items = (R)*.

### SEM: Measurement Model

The six-factor measurement model was examined with a CFA using maximum-likelihood estimation in AMOS (59). One item from the self-sacrifice scale and two items from the need for closure scale were dropped because their factor loadings were below 0.30. The CFA with all six constructs correlated provided a good fit to the data, $\chi_{\left(626\right)}^{2}$ = 1,153.04, *p* < 0.001, CFI = 0.94, TLI = 0.93, RMSEA = 0.04, SRMR = 0.07. The statistically reliable item loadings provide assurance that each latent variable is well-defined by its items.

#### SEM Full Model

The hypothesized model was tested by specifying a path linking social alienation to support for political violence and one path from the latter variable to wanting to join radical groups, controlling for collective narcissism, self-sacrifice, and need for closure. We display means, standard deviations, and correlations for all measures in Table 3. Results indicated that the hypothesized model fit the data well: $\chi_{\left(\text{df}=627,\text{N}=470\right)}^{2}$ = 1,153.32, *p* < 0.001, CFI = 0.94, TLI = 0.93, RMSEA = 0.04, SRMR = 0.07.

**Table 3:** Means, standard deviations, and correlations involving all variables from Study 1 (*N* = 470).

	***M***	***SD***	**2**	**3**	**4**	**5**	**6**
Social alienation (1)	2.27	1.03	0.59^***^	0.38^***^	^***^0.34	^***^0.24	−0.001
Support for political violence (2)	2.27	1.12		0.48^***^	0.38^***^	0.39^***^	0.04
Wanting to join a radical group (3)	2.89	1.47			0.37^***^	0.37^***^	0.04
Collective narcissism (4)	2.79	1.31				0.15^***^	0.23^***^
Self-sacrifice (5)	3.50	1.28					−0.01
Need for closure (6)	3.60	0.56					

^**^p < 0.01,

*** *p < 0.001*.

**Figure 1:**
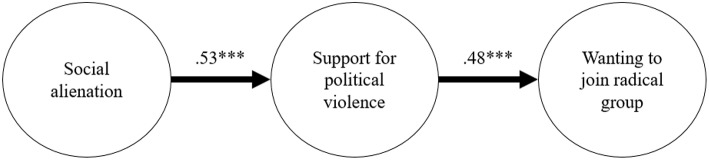
Results from Structural Equation Modeling (Study 1). For clarity, covariance paths and error terms are not shown. ^***^p < 0.001.

As shown in Figure 1, social alienation was positively related to support for political violence [*B* = 0.53, *SE* = 0.05, *t* = 9.17, *p* < 0.001; 95% CI = [0.43, 0.62]], which in turn was positively related to wanting to join a radical group (*B* = 0.48, *SE* = 0.07, *t* = 6.28, *p* < 0.001; 95% CI = [0.32, −0.61]). Self-sacrifice was related to both support for violence (*B* = 0.20, *SE* = 0.03, *t* = 6.51, *p* < 0.001; 95% CI = [0.14, 0.25]) and wanting to join a radical group (*B* = 0.18, *SE* = 0.04, *t* = 4.17, *p* < 0.001; 95% CI = [0.10, 0.25]). Collective narcissism was also positively related to political violence (*B* = 0.17, *SE* = 0.03, *t* = 4.91, *p* < 0.001; 95% CI = [0.11, 0.22]) and radical group (*B* = 0.13, *SE* = 0.04, *t* = 2.90, *p* < 0.001; 95% CI = [0.05, 0.20]). Need for closure, on the other hand, was not related to these variables (all *p*s > 0.05). Overall, the predictors explained 64 and 46% of the variance associated with support for political violence and wanting to join a radical group, respectively.

Indirect effects were investigated to further test the mediating role of support for political violence between social alienation and wanting to join a radical group. Consequently, bootstrapped confidence interval estimates of the indirect effect [see (62)] were calculated to confirm the significance of mediation. In the present study, the 95% confidence interval of the indirect effects was obtained with 5,000 bootstraps resamples (62). Results confirmed the hypothesized mediation (*B* = 0.25, *SE* = 0.05; 95% CI = [0.16, 0.37])^2^.

Overall, results from Study 1 demonstrated that the social alienation and support for political violence scales were psychometrically sound instrument. Furthermore, results provided correlational evidence supporting the 3N model of radicalization by demonstrating in a sample of Canadians that social alienation (Need) was positively associated with support for political violence (narrative), which in turn predicted wanted to join a radical organization (network). The question to which we turn next is whether these findings can be replicated cross-culturally.

## Study 2a-2b

The purpose of Study 2a and 2b was to adduce empirical evidence for the psychological trajectory postulated by the three Ns model of radicalization in different cultural contexts—an important step to demonstrate its external validity. We attempted to replicate Study 1 with a Pakistani sample (Study 2a) and a Spaniard sample (Study 2b) to demonstrate how social alienation, support for political violence, and radical social networks are interconnected, above and beyond the influence of other violence-related measures.

### Methods

#### Participants and Procedure

In Study 2a, assuming medium effect sizes, six latent variables, 12 observed variables, and power set at 0.80, a sample size of 400 was suggested (49). Four hundred and twenty-two Pakistani students (169 women; *M*_*age*_ = 20.70, *SD*_*age*_ = 2.11) participated in this research on a voluntary basis and completed a paper-and-pencil questionnaire. Verbal consent was obtained from participants. Similar analyses with 18 observed variables suggested a sample size of 200 participants. Two hundred and thirty-three Spaniards (165 women; *M*_*age*_ = 34.23, *SD*_*age*_ = 13.94) participated in this research on a voluntary basis and completed an online questionnaire. Written consent was obtained from participants.

### Materials

#### Social Alienation

In Study 2a, participants' feeling of social alienation was measured using two items from the social alienation scale (*r*_s_ = 0.52; *M* = 2.55, *SD* = 1.52) and three items in Study 2b (*M* = 2.32, *SD* = 1.21; α = 0.72).

#### Support for Political Violence

In Study 2a, participants' support for political violence was measured using two items from the support for violence scale (*r*_s_ = 0.60; *M* = 2.49, *SD* = 1.51) and three items in Study 2b (*M* = 1.86, *SD* = 1.41 *a* = 0.75).

#### Collective Narcissism

Collective narcissism was measured with two items in Study 2a (*r*_s_ = 0.47; *M* = 4.13, *SD* = 1.59) and three items in Study 2b (*M* = 3.28, *SD* = 1.50, *a* = 0.71).

#### Self-Sacrifice

Readiness to self-sacrifice was measured with two items in Study 2a (*r*_s_ = 0.47; *M* = 3.66, *SD* = 1.64) and three items in Study 2b (*M* = 2.74, *SD* = 1.47, *a* = 0.80).

#### Need for Closure

Need for closure was measured using two items in Study 2a (*r*_s_ = 0.59; *M* = 4.28, *SD* = 1.52) and three items in Study 2b (*M* = 4.47, *SD* = 1.45, *a* = 0.70).

#### Radical Social Network

The extent to which participants' social network is radical was measured using the following two items (Study 2a: *r*_s_ = 0.23; *M* = 3.17, *SD* = 1.53, Study 2b: *r*_s_ = 0.56; *M* = 2.28, *SD* = 1.46): “I personally know someone that supports violence for political change” and “People around me say it is appropriate to use violence for an ideology.” Participants rated their agreement to each of these items on a 7-point scale ranging from 1 (*not agree at all*) to 7 (very *strongly agree*).

### Results and Discussion

#### SEM: Measurement Model

The six-factor measurement model was examined with a CFA using maximum-likelihood estimation in AMOS (59). Expectation maximization (55) was used to replace isolated missing values (0.001% of all data) so that a covariance matrix based on the entire sample could be generated for the SEM analysis. The CFA with all six constructs correlated provided a good fit to the data, Study 2a $\chi_{\left(39\right)}^{2}$ = 63.67, *p* = 0.008, CFI = 0.95, TLI = 0.91, RMSEA = 0.03, SRMR = 0.03, Study 2b $\chi_{\left(104\right)}^{2}$ = 170.49, *p* < 0.001, CFI = 0.93, TLI = 0.92, RMSEA = 0.05, SRMR = 0.06. The statistically reliable item loadings provide assurance that each latent variable is well-defined by its items.

#### SEM Full Model

The hypothesized model was tested by specifying a path linking social alienation to support for political violence and one path from the latter variable to radical social network, controlling for collective narcissism, self-sacrifice, and need for closure. We display means, standard deviations, and correlations for all measures in Table 4. Results indicated that the hypothesized model fit the data well: Study 2a $\chi_{\left(39\right)}^{2}$ = 63.71, *p* = 0.01, CFI = 0.95, TLI = 0.92, RMSEA = 0.03, SRMR = 0.03, Study 2b $\chi_{\left(\text{df}=105\right)}^{2}$ = 175.11, *p* < 0.001, CFI = 0.93, TLI = 0.91, RMSEA = 0.05, SRMR = 0.06.

**Table 4:** Means, standard deviations, and correlations involving all variables from Study 2a (*N* = 422) and 2b (*N* = 233).

	***M***	***SD***	**2**	**3**	**4**	**5**	**6**
Social alienation (1)	2.72 (2.32)	1.55 (1.21)	0.31^***^ (0.25^***^)	0.21^***^ (0.15^*^)	0.13^**^ (0.06)	0.21^***^ (0.06)	0.02 (−0.03)
Support for political violence (2)	2.50 (1.86)	1.51 (1.41)		0.26^***^(0.18^**^)	0.12^**^(−0.02)	0.15^***^(0.09)	−0.07 (−0.00)
Radical social network (3)	3.17 (2.28)	1.54 (1.46)			0.10^*^ (0.05)	0.25^***^ (0.12^*^)	0.09 (−0.06)
Collective narcissism (4)	4.14 (3.28)	1.59 (1.50)				0.07 (0.24^***^)	0.12^*^ (0.17^**^)
Self-sacrifice (5)	3.67 (2.74)	1.64 (1.47)					0.06 (−0.07)
Need for closure (6)	4.29 (4.47)	1.51 (1.45)					

* *p < 0.05*,

** p < 0.01

*** *p < 0.001. Parameters of Study 2b are in parentheses*.

**Figure 2:**
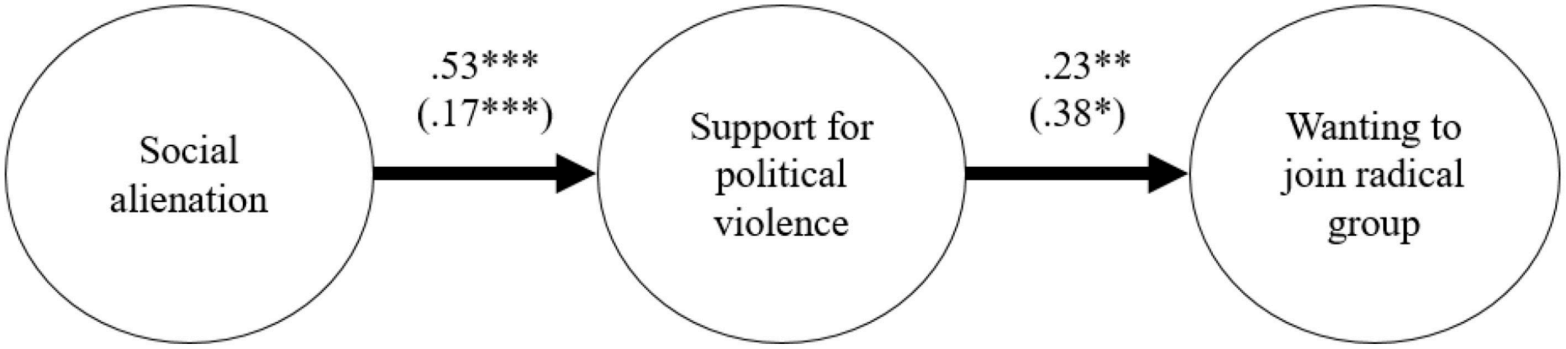
Results from Structural Equation Modeling (Study 2a in Pakistan and 2b in Spain). Parameters for Study 2b are in parentheses. For clarity, covariance paths and error terms are not shown. ^*^p < 0.05, ^**^p 0.01, ^***^p < 0.001.

As shown in Figure 2, social alienation was positively related to support for political violence (Study 2a: *B* = 0.53, *SE* = 0.13, *t* = 4.02, *p* < 0.001; 95% CI = [0.27, 0.78]; Study 2b: *B* = 0.17, *SE* = 0.05, *t* = 3.43, *p* < 0.001; 95% CI = [0.07, 0.26]), which in turn was positively related to knowing a radical social network (Study 2a: *B* = 0.23, *SE* = 0.07, *t* = 3.16, *p* = 0.002; 95% CI = [0.09, 0.36]; Study 2b: *B* = 0.38, *SE* = 0.18, *t* = 2.08, *p* = 0.03; 95% CI = [0.02, 0.73]). Self-sacrifice was not related to support for violence in Study 2a (*B* = 0.05, *SE* = 0.13, *t* = 0.36, *p* = 0.71; 95% CI = [−0.20, 0.30]), but it was significant in Study 2b (*B* = 0.09, *SE* = 0.03, *t* = 2.40, *p* = 0.01; 95% CI = [0.03, 0.14]). Self-sacrifice was related to radical social network in Study 2a (*B* = 0.17, *SE* = 0.05, *t* = 3.43, *p* < 0.001; 95% CI = [0.07, 0.26]), but not in Study 2b (*B* = 0.05, *SE* = 0.07, *t* = 0.69, *p* = 0.48; 95% CI = [−0.08, 0.18]). Collective narcissism and need for closure were not significantly related to any of the variables (Study 2a: all *p*s > 0.12; Study 2b all *p*s > 0.08). Overall, the explained variance associated with support for political violence was 34% in Study 2a (13% in Study 2b), whereas the explained variance associated with radical social network was 69% in Study 2a (8% in Study 2b).

Indirect effects were investigated to further test the mediating role of support for political violence between social alienation and radical social network. As in Study 1, bootstrapped confidence interval estimates of the indirect effect were calculated to confirm the significance of mediation. Results confirmed the hypothesized mediation (Study 2a: *B* = 0.12, *SE* = 0.06; 95% CI = [0.03, 0.31]; Study 2b: *B* = 0.06, *SE* = 0.05; 95% CI = [0.004, 0.21]).

Overall, Study 2a and 2b replicated Study 1 by demonstrating the relationship between social alienation, support for political violence, and radical social network, above and beyond the influence of other violence-related variables. Importantly, these correlational findings were replicated in one Western and one Asian country, suggesting that the 3N approach is relevant to the study of radicalization in different cultural contexts.

## Study 3

The purpose of Study 3 was to replicate Study 2 using an experimental design to test the causal link between social alienation and support for political violence. The aim of Study 3 was also to investigate the psychological mechanism that makes people feeling socially alienated more prone to support political violence. In this regard, the 3N model of radicalization postulates that moral disengagement is relevant to people adhering to violence (7). In his seminal work on moral disengagement, Bandura (14) discussed that violence could be seen as permissible when people engage in moral justification and dehumanization. We reasoned that if socially alienated people are detached from their community, then it follows that they should be more inclined to feel morally disengaged from other people, which should make them prone to support political violence. Consequently, we hypothesized that moral justification and dehumanization would mediate the relationship between social alienation and support for political violence.

### Methods

#### Participants and Procedure

Assuming medium effect sizes and power set at 0.80, a sample size of 225 people was suggested by 5,000 Monte Carlo simulations. Three hundred and fifty-seven Americans were recruited online via Amazon's Mechanical Turk. Duplicate IP addresses and geolocation were blocked using Turkprime service (64). Written consent was obtained from participants. Thirty-four participants did not complete the experimental manipulations and four did not successfully complete the attentional check embedded in the survey, thus leaving 319 participants (176 women; *M*_age_ = 40.09, *SD*_age_ = 13.02) for our analyses.

Participants were randomly assigned to one of two experimental conditions. The social alienation condition consisted of a recall task developed using two items taken from the social alienation scale [for a similar procedure see (65, 66)]. Specifically, participants (*N* = 130) were instructed to:

“Write about a time when you tried to distance yourself from the average American and you did not strongly identify with American culture and values. Recall this event vividly and include as many details as you can to relive the experience—the whole story.”

Participants in the control condition (*N* = 189) were instructed to:

“Write about a time when you experienced dental pain and it took a long time to go away. Recall this event vividly and include as many details as you can to relive the experience—the whole story.”

### Materials

#### Social Alienation

Participants' feeling of social alienation was measured as in Study 1 (α = 0.82; *M* = 2.86, *SD* = 1.18), excluding the two items used in social alienation manipulation.

#### Moral Disengagement

Participants' moral disengagement was measured using a short 7-item version of Bandura et al.'s (38) scale. A factor analysis using oblimin rotation and maximum likelihood were conducted on these items. As expected the scree test and eigenvalues (4.15 and 1.06) indicated the extraction of two factors explaining 59 and 15% of the variance, respectively. The first factor was composed of items measuring dehumanization (e.g., “Some people deserve to be treated like animals”; α = 0.90; *M* = 2.06, *SD* = 1.40), whereas the second factor measured moral justification (“It is alright to fight when your group's honor is threatened”; α = 0.66; *M* = 3.51, *SD* = 1.59). Participants rated their agreement to each of these items on a 7-point scale ranging from 1 (*not agree at all*) to 7 (*very strongly agree*).

#### Support for Political Violence

Participants' support for political violence was measured using the same scale as in Study 1 (*a* = 0.85; *M* = 2.32, *SD* = 1.20).

### Results and Discussion

Our first analysis tested the influence of the experimental manipulation on the social alienation measure, which consisted of our manipulation check. Results indicated that compared to the control group (*M* = 2.64, *SD* = 1.08), participants in the experimental condition (*M* = 3.19, *SD* = 1.24) reported greater social alienation, *F*_(1, 317)_ = 17.84, *p* < 0.001, η ^2^ = 0.05. The experimental condition also had an impact on support for political violence: compared to the control group (*M* = 2.21, *SD* = 1.15), participants in the experimental condition (*M* = 2.47, *SD* = 1.26) reported greater support for political violence, *F*_(1, 317)_ = 3.69, *p* = 0.05, η ^2^ = 0.01.

In our second analysis, we tested the mediating role of moral justification and dehumanization between the experimental conditions (coded 0 = control group; 1 = social alienation) and support for political violence using path analysis. We display means, standard deviations, and correlations for all measures in Table 5.

**Table 5:** Means, standard deviations, and correlations involving all variables from Study 3 (*N* = 319).

	***M***	***SD***	**2**	**3**	**4**	**5**
Experimental condition (1)	0.40	0.49	0.23^***^	0.10^*^	0.11^*^	0.04
Social alienation (2)	2.86	1.18		0.28^***^	0.05	0.24^***^
Support for political violence (3)	2.32	1.20			0.37^***^	0.59^***^
Moral justification (4)	3.51	1.59				0.48^***^
Dehumanization (5)	2.06	1.40				

* *p < 0.05*,

*** *p < 0.001*.

Results indicated the model had an acceptable fit to the data $\chi_{\left(1\right)}^{2}$ = 2.39, *p* = 0.12, CFI = 0.99, TLI = 0.96, RMSEA = 0.06, SRMR = 0.02. Results indicated that the experimental manipulation did not influence dehumanization (*B* = 0.13, *SE* = 0.16, *t* = 0.85, *p* = 0.39; 95% CI = [−0.18, 0.44]), but did have an impact on moral justification (*B* = 0.37, *SE* = 0.18, *t* = 2.05, *p* = 0.04; 95% CI = [0.01, 0.72]), explaining 1.3% of the variance, such that participants in the social alienation condition (*M* = 3.73, *SD* = 1.60) reported greater moral justification than those in the control condition (*M* = 3.36, *SD* = 1.56). In turn, both dehumanization (*B* = 0.46, *SE* = 0.04, *t* = 10.66, *p* < 0.001; 95% CI = [0.38, 0.53]) and moral justification (*B* = 0.08, *SE* = 0.03, *t* = 2.20, *p* = 0.02; 95% CI = [0.02, 0.13]), were positively associated with support for political violence, explaining 36% of its variance.

**Figure 3:**
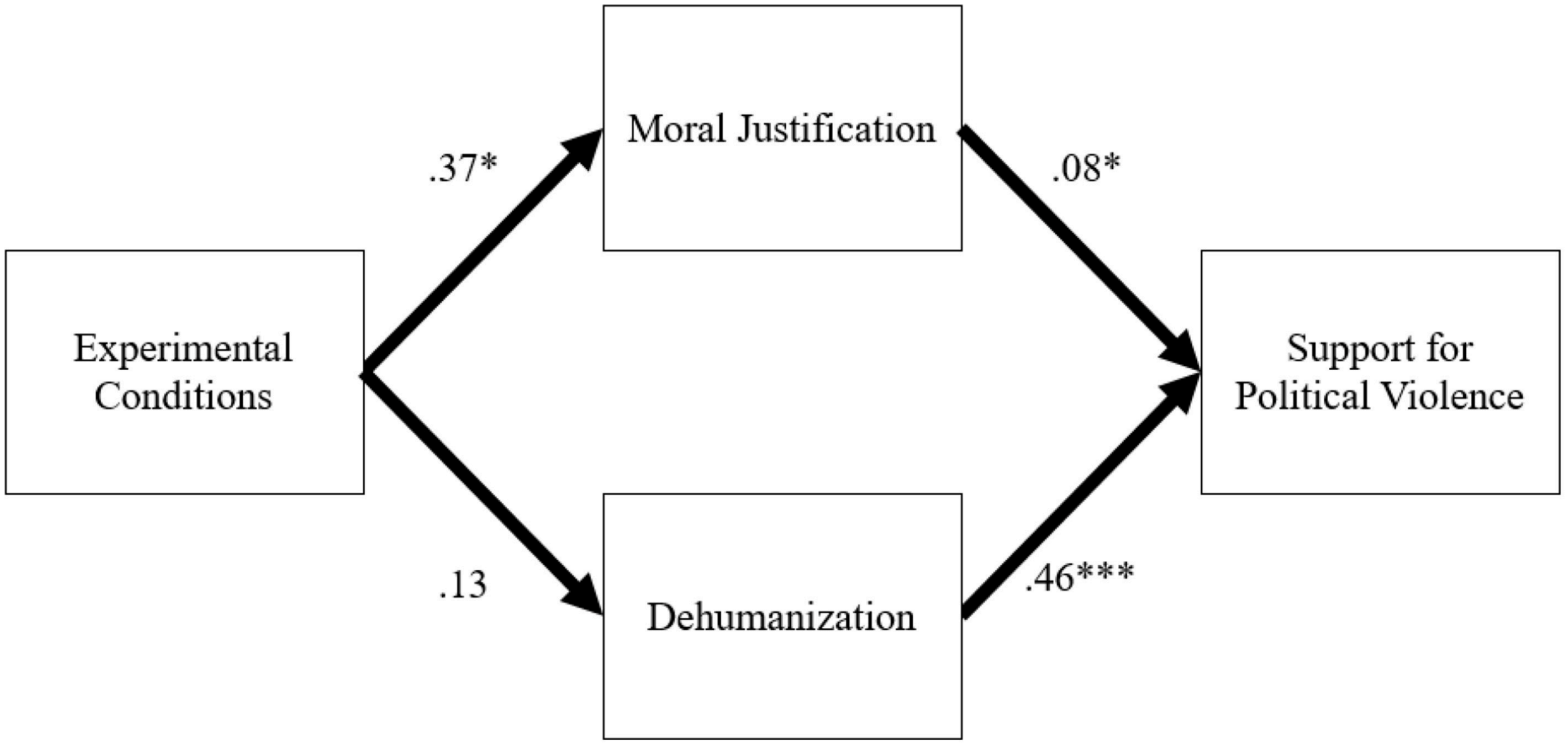
Results from Path Analysis (Study 3). For clarity, covariance paths and error terms are not shown. Experimental Condition: 0 = Control group; 1 = Social Alienation. ^*^p < 0.05, ^***^p < 0.001.

Bootstrapped bias-corrected confidence interval estimates [see (62)] were calculated to test the mediating role of moral justification between the experimental condition and support for political violence. Results confirmed the significance of the mediation (*B* = 0.03, *SE* = 0.02; 95% CI = [0.00, 0.10]; see Figure 3).

Results of Study 3 suggest that social alienation has a causal influence on people's support for political violence. Importantly, this relationship was mediated by people's proclivity to morally justify the use of violence, which was also enhanced by the experimental manipulation. Dehumanization, on the other hand, also predicted people's support for political violence, but the situational induction of social alienation did not make people more prone to dehumanize others. This hints to the possibility that moral justification comes into play first in people's trajectory toward violence, whereas dehumanization potentially comes at a later stage in the radicalization process. Of note, one limitation associated with Study 3 is that the reported effect sizes were relatively weak. For example, the effect of the experimental manipulation on moral justification only explained 1.3% of the variance and the corresponding mediation effect on support for violence had a confidence interval with a lower bound close to zero. Overall then, although consistent with Study 1 and 2, Study 3's results would benefit from being replicated.

## General Discussion

A fuller understanding of why and how individuals radicalize and commit acts of terrorism merits immediate attention. According to the 3N model of radicalization (12), there are three major components that push individuals toward violent extremism: (1) the Need element which pertains to individuals' quest for personal significance [see (7, 13, 17)], (2) the Narrative which identifies the means to the end of significance; in a violence justifying narrative this is portrayed as extreme aggression against perceived enemies (ethnic, religious, or social), and (3) the social Network in which individuals are embedded and that validates the means-ends relations between violence and significance as well as dispensing rewards (in terms of bestowed status and veneration) to people that commit violence.

To test the theoretical postulates of the 3N model, we developed two new psychometric instruments: the social alienation scale and the support for political violence scale. Items for both scales were constructed by the research team and then presented to SMEs, which included experts from several key areas including academia, extremism policing, Canadian Border Services Agency intelligence, Canadian Security Intelligence Service, Central Intelligence Agency, and Canadian Special Forces. One SME was an ex-radical turned CSIS agent. Exploratory and confirmatory factor analyses confirmed that the scales were psychometrically sound.

Using these scales, the present research adduced empirical evidence to support the 3N model of radicalization, thus yielding practical and theoretical implications. Study 1 demonstrated that social alienation (Need) was positively related to support for violence (Narrative), which in turn was positively associated with wanting to join a radical group (Network), controlling for other predictors known to be related to violence, namely collective narcissism, need for closure, and self-sacrifice.

In Studies 2a and 2b, we replicated Study 1 in two culturally distinct samples, namely, in Pakistan and in Spain. Consistent with our theoretical framework, we found that socially alienated individuals reported greater support for political violence, which in turn was positively associated with knowing like-minded individuals that support violence. These results demonstrated that the 3N model of radicalization is robust and has good external validity.

In Study 3, we replicated Study 1, 2a, and 2b using an experimental design with yet another culturally distinct group of participants (i.e., Americans) and demonstrated the causal link between social alienation and support for political violence. An important theoretical contribution of this study consisted of demonstrating that moral justification (but not dehumanization) mediated the relationship between the social alienation manipulation and support for political violence. Prior theorizing related to the 3N model of radicalization acknowledged that ideological narratives containing cognitive maneuvers allowing unethical behaviors to be carried out without self-recrimination would morally warrant the use of political violence (7). In this research, we found empirical support for this proposition and demonstrated that Need (i.e., social alienation) and Narrative (i.e., support for political violence) are connected through this psychological mechanism. Future research could examine if other forms of significance quest activation (e.g., feeling incompetent, lack of meaning in life), which are not necessarily associated with feeling detached from society, also result in moral disengagement. It could well be that only a certain subset of situations that arouse the significance quest produces that phenomenon. Understanding under which circumstances moral disengagement occurs (or not) would be an important theoretical advancement.

Overall, the results herein described lend support to the 3N model of radicalization and illustrates how Need, Narrative, and Network are sequentially interconnected. This is not to say that there are no other possible radicalization trajectories—alternative trajectories most certainly exist—but this is the first to be empirically tested and supported. For example, some trajectories could initially involve having bonds, or even familial ties, with individuals that support violence (Network), which would increase the likelihood that a person adheres to the group's ideology (Narrative) and its collective grievance (Need). In other cases, the journey to violent extremism could begin with exposure to the ideological narrative (e.g., on social media), followed by a sense of grievance and disgruntlement (Need), which would culminate in wanting to join the radical group (Network). Interestingly, one could hypothesize that some of these trajectories would be more prevalent depending on the social context. For example, one would expect that in collectivistic (vs. individualistic) societies the role of social networks would play a more important role in the first steps en route to violent extremism. More research is needed to examine these questions.

## Limitations

The present work is not impervious to methodological limitations. First, most of the research presented here (except Study 3) is correlational, which limits the use of causal inferences to describe the relationship between Need, Narrative, and Network. Additional experimental evidence is needed to make these claims. Second, although we have accumulated cross-cultural evidence for the 3N model, the present research does not entirely demonstrate how the process of radicalization unfolds over time. Consequently, future research should gather longitudinal data to assess within-subject changes in support for political violence and radical social network to relate these changes to individuals' experience of social alienation. Third, the present research has focused exclusively on the risk factors associated with political violence; a more comprehensive analysis of radicalization would necessitate investigating protection factors such as the presence of peaceful alternatives to further one's political goal, empathy, emotion regulation, and strong familial ties. Fourth, instead of relying on self-report measures, future research could also include behavioral outcomes, including implicit measures (e.g., IAT, lexical decision task), to observe the relevance of social alienation for political violence. Last, but not the least, we believe that objective measures of radical social network should also be used in future research. For example, participants' social media connections and communications could be utilized to measure people's ties to radical milieus (67).

## Conclusion

The present research examined a radicalization trajectory proposed by the 3N model of radicalization whereby individuals transition from losing significance (feeling socially alienated), to adhering to violence-justifying ideologies, to wanting to join radical groups. In addition to finding empirical evidence for this model across four culturally distinct samples, this research showed that moral justification is one of the mechanisms linking social alienation to support for political violence. These findings extend our knowledge on the psychology of radicalization and provide additional knowledge for frontline workers (e.g., social workers, psychologists, family counselors) to identify and ultimately prevent individuals from engaging in violent extremism. Efforts to prevent terrorism need to start with reliable data that can both guide future research and enlighten stakeholders to draw informed conclusions. Our research provides theory-driven findings to help researchers, practitioners, and policy-makers to develop community-based efforts to reach these goals (68).

## Ethics Statement

(1) Chesapeake Institutional Review Board, Protocol number: Pro00012629. (2) New York University Abu Dhabi, Protocol number: 043-2017. Written consent was obtained from human subjects in Study 1, 2b, and 3. Verbal consent was obtained from participants in Study 2a.

## Author Contributions

JB, LR, M-AL, PM, and KF design and Interpretation of results: original idea and theorizing, planning and input, supervisory role: active supervision of the project, data acquisition: original experimental work, data analysis, writing: drafting, revising, and final approval of the article to be published. MM, HM, and NN design and Interpretation of results: original idea and theorizing, planning, and input, writing: drafting, revising, and final approval of the article to be published.

### Conflict of Interest Statement

The authors declare that the research was conducted in the absence of any commercial or financial relationships that could be construed as a potential conflict of interest.

## Footnotes

^1^We would like to thank the following subject matter experts for helping with creating the scales: Dr. Elaine Pressman, Senior Fellow in the Canadian Centre of Intelligence and Security Studies at Carleton University. Dr. Robert Barrett - Security and foreign affairs specialist Detective John Byers - Ottawa Police Service intelligence officer Dr. Stéphane Dandeneau - Professor of psychology Dan Dunlop - Retired OPS detective and hate crime pioneer Phil Gurski - Retired CSIS strategic analyst D.L. - Retired Canadian special forces Dr. Juan Rivera - Retired Central Intelligence Agency senior operative Mubin Shaikh - Previous supporter of the militant jihadi culture Warren Silver - National training officer for police services Dr. Anne Speckhard - World-renowned researcher on terrorism Alison Vermette - Canadian Border Service Agency intelligence officer Anders D. Melchiorsen, Lead intelligence-analysis liaison officer for the Danish National Police in Denmark.

^2^As discussed by Thoemmes (63), testing alternative models “to check whether one mediation model is superior to another is inadmissible” (p. 1). Therefore, these tests were not conducted.
